# Nerves in Bone: Evolving Concepts in Pain and Anabolism

**DOI:** 10.1002/jbmr.3822

**Published:** 2019-07-26

**Authors:** Jennifer M Brazill, Alec T Beeve, Clarissa S Craft, Jason J Ivanusic, Erica L Scheller

**Affiliations:** ^1^ Department of Internal Medicine, Division of Bone and Mineral Diseases Washington University St. Louis MO USA; ^2^ Department of Biomedical Engineering Washington University St. Louis MO USA; ^3^ Department of Cell Biology and Physiology Washington University St. Louis MO USA; ^4^ Department of Anatomy and Neuroscience University of Melbourne, Melbourne Victoria Australia

**Keywords:** BONE‐BRAIN‐NERVOUS SYSTEM INTERACTIONS, SYSTEMS BIOLOGY ‐ BONE INTERACTORS, OTHER, ANALYSIS/QUANTITATION OF BONE, OTHER, THERAPEUTICS, ANABOLICS, THERAPEUTICS

## Abstract

The innervation of bone has been described for centuries, and our understanding of its function has rapidly evolved over the past several decades to encompass roles of subtype‐specific neurons in skeletal homeostasis. Current research has been largely focused on the distribution and function of specific neuronal populations within bone, as well as their cellular and molecular relationships with target cells in the bone microenvironment. This review provides a historical perspective of the field of skeletal neurobiology that highlights the diverse yet interconnected nature of nerves and skeletal health, particularly in the context of bone anabolism and pain. We explore what is known regarding the neuronal subtypes found in the skeleton, their distribution within bone compartments, and their central projection pathways. This neuroskeletal map then serves as a foundation for a comprehensive discussion of the neural control of skeletal development, homeostasis, repair, and bone pain. Active synthesis of this research recently led to the first biotherapeutic success story in the field. Specifically, the ongoing clinical trials of anti‐nerve growth factor therapeutics have been optimized to titrated doses that effectively alleviate pain while maintaining bone and joint health. Continued collaborations between neuroscientists and bone biologists are needed to build on this progress, leading to a more complete understanding of neural regulation of the skeleton and development of novel therapeutics. © 2019 The Authors. *Journal of Bone and Mineral Research* published by Wiley Periodicals, Inc.

## Development of a Unified Theory for Nerves in Bone

**Figure 1:**
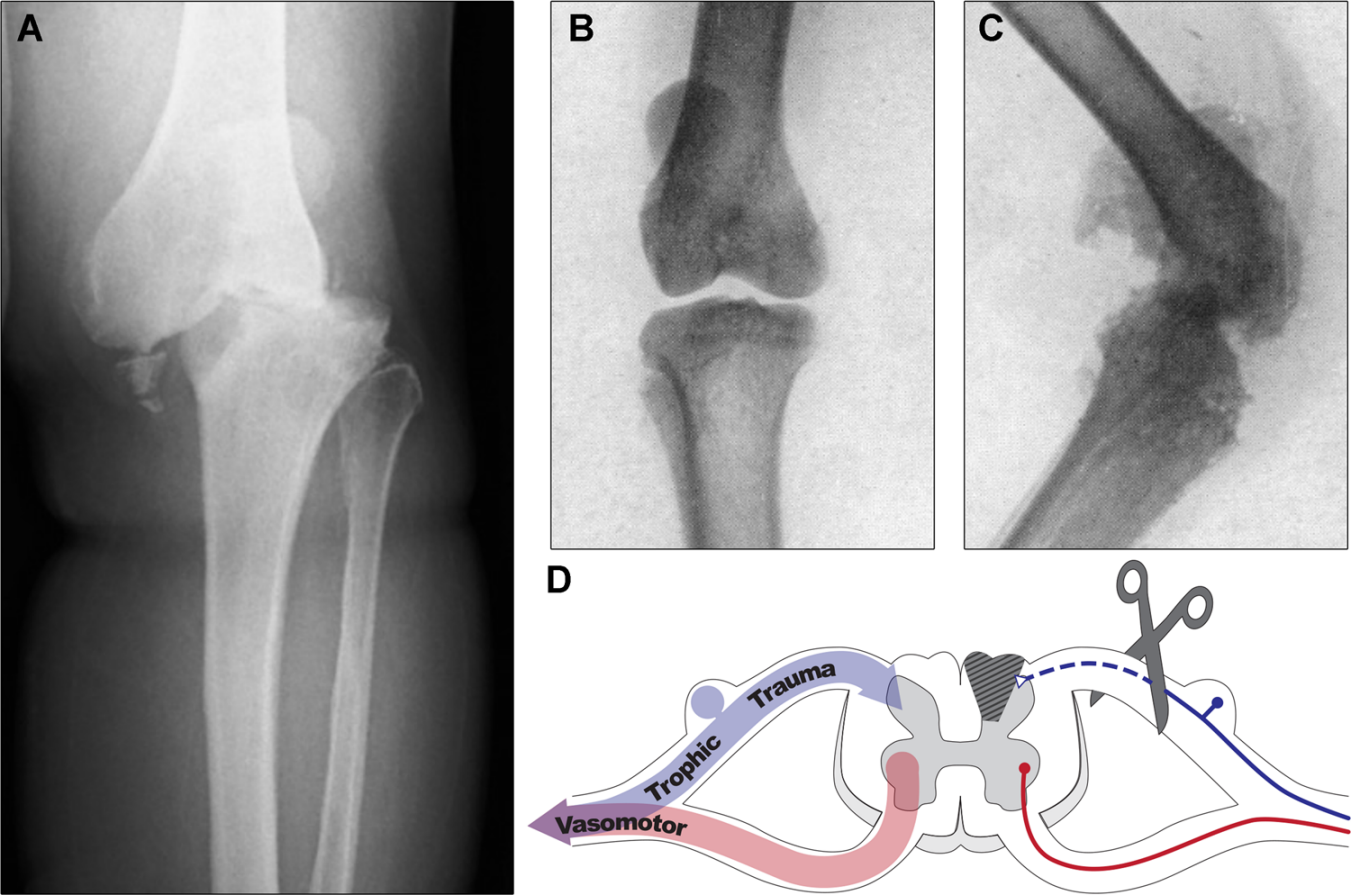
Multifactorial neural regulation of bone and joint. (*A*) Anteroposterior X‐ray of the knee joint of a 62‐year‐old patient with unilateral Charcot neuroarthropathy with characteristic joint collapse, bone loss, and fragmentation. Patient presented with chronic polyneuropathy and total sensorineural loss following a failed spinal stenosis surgery. Reprinted with permission from Cıvan and colleagues.^182^ (*B*, *C*) X‐ray of the knee joints of Eloesser's cat showing a frontal projection of the control side (*B*) and lateral view of the affected side (*C*); the affected knee was subjected to an acute thermocautery trauma following sensory posterior root resection as depicted in *D* (scissors) and subsequently developed deformity and grating of joint surfaces within 3 weeks. Reprinted with permission from Eloesser.^3^ (*D*) Conceptual representation of neurotrophic, neurotraumatic, and neurovascular theories of neural regulation of bone and joint, where sympathetic nerves regulate vascular tone (red), and sensory nerves provide trophic signals and mediate protective pain perception, as well as vasoregulation (blue). Collectively, the peripheral nerve carries different types of neurons that contribute to bone and joint homeostasis; destruction of these, eg, upon dorsal column degeneration (gray hatched region of spinal cord) with tabes dorsalis or dorsal root resection (scissors) and degeneration of the central primary sensory axon, contributes to joint collapse and bone loss.

The field of skeletal neurobiology emerged with a great debate surrounding the relationship between sensory nerve damage and joint disease that began in the mid‐19th century. In 1868, French neurologist Jean‐Martin Charcot detailed joint pathology with progressive degeneration of bones and soft tissues in patients with tabes dorsalis, a complication of untreated syphilis resulting in degeneration of sensory nerves in the dorsal column of the spinal cord^1^ (Fig. 1 *A*, *D*). Charcot speculated that nerves in bone were of a trophic nature. He reasoned that their destruction disrupted the supply of growth factors to the bone and joint, leading to their collapse. Thus, Charcot laid the foundation for the neurotrophic theory of bone and joint health. Almost immediately, this was met with opposition by the neurotraumatic theorists, led by Volkman and Virchow, who asserted that nerve damage results in loss of peripheral sensation leading to repetitive trauma that outpaces healing. This theory was supported by a series of sensory denervation experiments performed in cats by Eloesser in 1917 and Corbin and Hinsey in 1939. They concluded that loss of sensation alone was insufficient to induce neuropathic osteoarthropathy, since only denervated cats exhibiting unnatural gait and inappropriate loading developed bone and joint lesions^2^, ^3^ (Fig. 1 *B*–*D*). In the 1980s, a third theory emerged following the observation that blood flow was increased in joints of patients with diabetic neuropathy.^4^ This neurovascular theory was based on the sympathetic control of vascular tone, which when compromised by autonomic neuropathy, allows excess blood flow to affected joints leading to inflammation‐induced bone resorption and susceptibility to minor trauma.

Now, after more than a century of investigation, the collective model points to a multifactorial relationship between nerves and bone which synthesizes the neurotrophic, neurotraumatic, and neurovascular theories (Fig. 1 *D*). Indeed, trophic signals and protective pain, as well as regulation of blood flow, are all indispensable components of neural regulation of bone and joint. In recognition of this synergy, this review will first summarize what is known regarding the neuronal subtypes found in the skeleton, their distribution within bone compartments, and their central projection pathways. This neuroskeletal map will then guide a comprehensive discussion of the neural control of skeletal development, homeostasis and repair, and last, bone pain.

## Classification of Skeletal Axons

**Figure 2:**
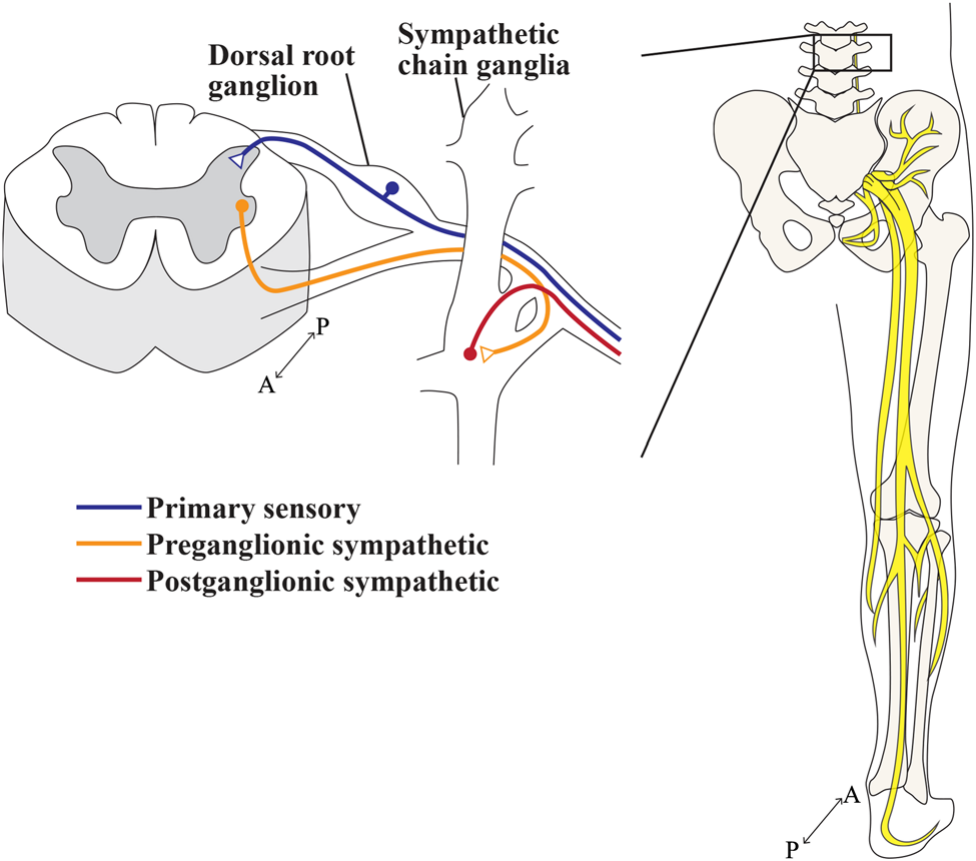
Peripheral neurons’ route to the skeleton. A cross‐section of the lumbar spinal cord, where peripheral neurons that innervate the lower limbs either originate or terminate. Primary sensory neurons (blue) are pseudo‐unipolar: cell bodies reside in dorsal root ganglia from which a single axonal process bifurcates into a centrally projecting axon that targets the dorsal horn of the spinal cord and a long peripheral axon that projects out to the target tissue. The sympathetic system communicates to peripheral targets via a two‐neuron relay. Preganglionic sympathetic axons (orange) originate in the intermediolateral cell column of the spinal cord and project to sympathetic ganglia, mostly the sympathetic chain ganglia for innervation of the lower limb, to synapse with postganglionic sympathetic neurons. Postganglionic sympathetic neurons (red) project long axons towards their targets. The sensory and sympathetic axons are carried through major mixed nerves, as depicted for the right lower limb in relation to the skeleton, posterior view. Proximal to the bone, fine nerve branches containing the small sensory and autonomic axons described leave to supply the periosteum, bone, and bone marrow.

**Figure 3:**
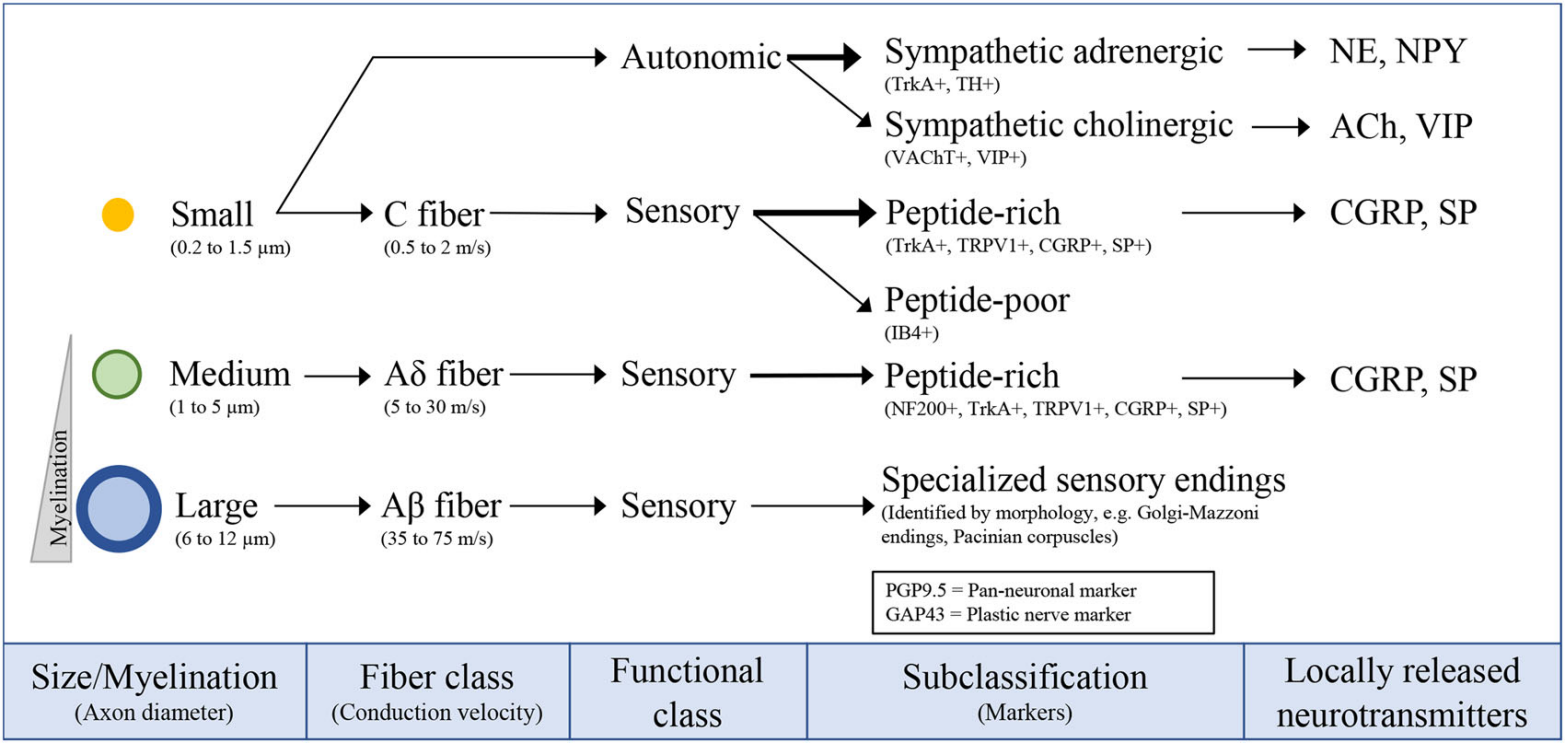
Classification of skeletal axons. The axons in peripheral nerves supplying the bone can be classified on the basis of their size and myelination status, both of which contribute to conduction velocity.^14^ The majority of skeletal fibers are unmyelinated, small diameter axons (yellow, 0.2 to 1.5 µm) that conduct slowly (0.5 to 2 m/s). These consist of sympathetic fibers, which can have either an adrenergic or cholinergic phenotype, and sensory C fibers. The second most prevalent axon type found in the bone are Aδ fibers: lightly myelinated, medium diameter axons (green, 1‐µm to 5‐µm diameter) that conduct at medium speeds (5 to 30 m/s). Last, Aβ fibers are large‐diameter, thickly myelinated axons (6 to 12 µm) that conduct much faster (35 to 75 m/s) but are relatively scarce or absent in bone. Each fiber subclass has a unique set of markers that are often used to define it immunohistochemically. Small‐sized and medium‐sized neurons of both sensory and/or autonomic origin also have the capacity to release specific neurotransmitters to the local environment. TrkA = tyrosine kinase receptor type 1; TH = tyrosine hydroxylase; VAChT = vesicular acetylcholine transporter; VIP = vasoactive intestinal peptide; TRPV1 = transient receptor potential cation channel subfamily V member 1; CGRP = calcitonin gene‐related peptide; SP = substance P; IB4 = isolectin‐B4; NF200 = neurofilament 200; PGP9.5 = protein gene product 9.5; GAP43 = growth associated protein 43; NE = norepinephrine; NPY = neuropeptide Y; ACh = acetylcholine.

The peripheral nervous system (PNS) relays information between the central nervous system (CNS) and the skeleton. Neurons of the PNS are located in sensory and autonomic ganglia, collections of neural cell bodies that disseminate their peripheral axons to target tissues through nerve bundles (eg, sciatic nerve, tibial nerve, etc) (Fig. 2). The consensus in the field is that the fine nerve branches that target the skeleton are primarily composed of small and medium diameter axons, both unmyelinated and myelinated.^5^, ^6^, ^7^, ^8^, ^9^, ^10^ These morphological parameters are consistent with primary sensory neurons and postganglionic autonomic neurons and have been further classified by neurochemical profiling and electrophysiological properties as discussed below (Fig. 3).

### Sensory nerves in bone

Primary sensory neurons in bone house their cell bodies in dorsal root ganglia (DRG) alongside the spinal cord (Fig. 2), or cranial nerve ganglia for those that innervate the craniofacial skeleton. They have a pseudo‐unipolar morphology, meaning that the neuron extends one axon which bifurcates into a central projection toward the brain and a peripheral projection which innervates the target tissue. This connects their receptive fields in the periphery to the dorsal horn of the spinal cord for central processing of pain, pressure, and other perceived stimuli. In addition, these neurons can generate efferent signals, often in the form of dorsal root reflexes, that are directed *toward* their peripheral targets. In general, this antidromic activity can arise from collateral axon branches within or proximal to the target tissue, from the dorsal root ganglia, or from central or local circuits in the dorsal horn.^11^ The efferent activity of primary sensory neurons is most evident in neurogenic inflammation following tissue injury, where release of neuropeptides such as calcitonin gene‐related peptide (CGRP) and substance P (SP) promotes vasodilation and plasma extravasation, respectively.^12^ The presence of neurogenic inflammation within bone is supported by a study in rats in which chemical sensory denervation suppressed vasodilation and inflammatory cell recruitment within the bone marrow after induction of adjuvant‐induced arthritis.^13^ This capacity for local neuropeptide release also speaks to the trophic nature of sensory nerves in bone, elaborated below in the Molecular Signals From Neurons to Bone‐Neurotransmitters section.

Primary sensory neurons are categorized by size, myelination status, neuropeptide content, growth factor dependence, and conduction velocity (Fig. 3).^14^ Peptide‐rich fibers expressing CGRP and SP are prevalent in bones across vertebrate species.^5^, ^7^, ^10^, ^15^, ^16^, ^17^ These exist as both small unmyelinated (0.2 to 1.5 µm) or medium myelinated fibers (1 to 5 µm). The myelinated axons are generally identified by their immunoreactivity for the high molecular weight (200 kD) neurofilament protein NF200.^10^ Peptide‐poor C fibers have not yet been detected within bone,^10^, ^18^ but isolectin‐B4 (IB4)‐binding has been demonstrated in 11% to 34% of DRG neural cell bodies innervating the rat tibia, suggesting that they may represent a larger population than previously recognized.^19^ Survival and recruitment of peptidergic fibers is nerve growth factor (NGF)‐dependent and peptide‐poor fibers are glial‐derived neurotrophic factor (GDNF)‐dependent.^20^, ^21^ Upon noxious mechanical stimulation, conduction velocities recorded from fine nerves innervating the rat bone are consistent with small, unmyelinated C fibers (<2 m/s) and medium sized, myelinated Aδ axons (2 to 12.5 m/s),^22^ which are well matched with reported conduction velocities of somatic sensory afferents (C fibers: 0.5 to 2 m/s; Aδ fibers: 5 to 30 m/s; Aβ fibers: 35 to 75 m/s).^14^ Large‐diameter neurons are generally absent within bone marrow; however, large sensory fibers with encapsulated endings specialized for detecting vibration and pressure have been described in the interosseous membranes between bones,^23^ in the jaws anterior to the mental foramen in cats,^24^ and in the human long bone periosteum.^25^

### Autonomic nerves in bone

The sympathetic and parasympathetic divisions of the autonomic nervous system send messages to the periphery through a two‐neuron relay. Autonomic outflow is communicated through a preganglionic neuron in the spinal cord that synapses onto the cell body of a postganglionic neuron within an autonomic ganglion (Fig. 2); this largely occurs in the sympathetic chain ganglia for the trunk and the cervical ganglia for the head. Both divisions send small, unmyelinated neurons to peripheral targets. Postganglionic autonomic neurons have slow conduction velocities, in the range of C fibers, and are similarly small in size and devoid of myelination (Fig. 3). The autonomic nervous system coordinates involuntary functions in response to internal and external stressors to maintain whole‐body homeostasis, including vascular tone, and is always active at a basal level. Energy expenditure, circadian clock rhythms, and stress are just a few possible signals that have been shown to influence sympathetic outflow with subsequent impacts on bone metabolism.^26^, ^27^, ^28^

Postganglionic autonomic neurons are identified by the presence of cognate small molecule neurotransmitters or enzymes. Sympathetic adrenergic neurons expressing tyrosine hydroxylase (TH), the rate‐limiting enzyme for synthesis of norepinephrine (NE), are abundant in bone.^7^, ^8^, ^10^, ^15^, ^16^, ^17^, ^29^ Cholinergic neurons containing acetylcholine (ACh), as evidenced by expression of vesicular ACh transporter (VAChT), and the neuropeptide vasoactive intestinal peptide (VIP), also innervate bone.^7^, ^8^, ^16^, ^30^, ^31^ Although cholinergic fibers in the periphery are mostly derived from parasympathetic postganglionic neurons, there is in fact a subset of postganglionic sympathetic neurons that also have a cholinergic neurochemical profile.^30^, ^32^, ^33^ For example, in the periosteum, a subset of sympathetic neurons acquires a cholinergic phenotype upon contact with their target.^31^, ^34^ Furthermore, sympathectomy abolishes VIP staining in bone,^8^, ^35^ supporting a sympathetic origin for the VIP + cholinergic fibers targeting the skeleton. At present, evidence for a parasympathetic innervation of the bone is lacking (there is no evidence for direct tracing from autonomic postganglionic nerves in bone to parasympathetic ganglia).^36^

### Other biomarkers of both neuronal and non‐neuronal cells in bone

Several additional biomarkers often appear in the skeletal neurobiology literature. First, neurotrophic tyrosine kinase receptor type 1 (TrkA) is a member of the Trk receptor family and one of two cognate receptors for NGF. Many peptidergic sensory nerves and the majority, if not all, TH + autonomic neurons in bone express TrkA.^37^ This implies that axon guidance, survival, sprouting, and activity of these neurons could be influenced by NGF signaling.^37^ Vascular endothelial cells, periodontal fibroblasts, and osteoblasts have also been reported to express TrkA.^38^, ^39^, ^40^, ^41^ Second, the vast majority of small sensory and autonomic nerve fibers express growth associated protein 43 (GAP43), a protein that has been related to an axon's capacity for growth, regeneration, and plasticity.^29^, ^37^, ^42^ GAP43 is also expressed by non‐myelinating Schwann cells.^43^ Third, the transient receptor potential cation channel subfamily V member 1 (TRPV1) is expressed by most unmyelinated and some small diameter myelinated sensory neurons, including those in bone and joint.^44^, ^45^, ^46^ TRPV1 + neurons in bone can be sensitized by the TRPV1 agonist capsaicin, a potent extract of the chili pepper.^22^ High systemic doses of capsaicin have also been used to induce cell death selectively in small sensory, primarily unmyelinated, neurons in bone and other tissues.^13^, ^47^ Though originally thought to be specific to nerve, recent reports have suggested that TRPV1 is also expressed in cells of the chondrocyte, osteoblast, and osteoclast lineages.^48^, ^49^ Last, protein gene product 9.5 (PGP9.5) is a ubiquitin‐protein hydrolase that is often used as a pan‐neuronal marker for purposes of quantification of nerves in and on the bone.^29^, ^42^, ^50^

## Nerve Entry and Distribution Within the Skeleton

**Figure 4:**
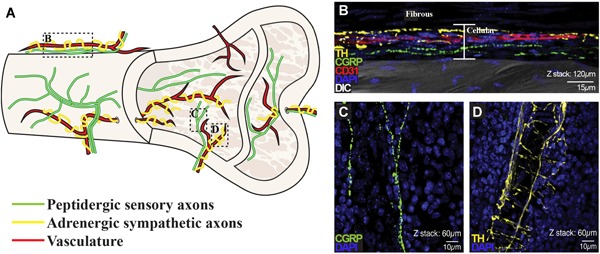
Distribution and patterning of nerves in bone. (*A*) Schematic of the peptidergic sensory axons and adrenergic sympathetic axons, highlighting their relationship to the vasculature, as well their relative distributions and typical patterning in the periosteum, as they enter the cortical bone, and within the marrow space. (*B*–*D*) Representative confocal micrographs of the distal mouse femur with immunostaining for TH + sympathetic fibers (yellow), CGRP + peptidergic sensory fibers (green), CD31 + endothelial cells of blood vessels (red), showing cell nuclei (DAPI, blue) and overlaid on an image captured by DIC microscopy to provide orientation in the periosteum (*B*) and marrow (*C*, *D*). *Z*‐stack thickness and scale bars as indicated. *B*–*D* reprinted and adapted with permission from Chartier and colleagues.^15^ DIC = differential interference contrast.

Sensory, and less so autonomic, nerves form subtype‐specific meshwork patterns within the periosteum that descend through the outer connective tissue layers and inner cellular layers into the bone itself^7^, ^10^, ^17^ (Fig. 4 *A*, *B*). Sensory and autonomic fibers penetrate the cortical bone alongside vessels through nutrient canals in mice and through Volkmann's canals and Haversian systems in humans, cats, and other large vertebrates.^51^, ^52^, ^53^ In the distal femur of an adult mouse, 26% of the nutrient canals are occupied by CGRP + sensory axons whereas nearly double the proportion of canals contain TH + sympathetic fibers.^15^ Within the canals, sensory nerves are generally linear, whereas sympathetic fibers spiral around vessels, reminiscent of perivascular patterning in the periosteum. Once in the marrow space, unmyelinated CGRP + sensory fibers branch and project linear, varicose‐rich endings, particularly in the vicinity of the epiphyseal trabecular bone, and less frequently in the metaphysis and diaphysis^10^, ^17^ (Fig. 4 *A*, *C*). Myelinated NF200 + sensory axons, most of which also contain CGRP, share a similar pattern of distribution, but tend to have a relatively longer and more linear morphology.^10^ Small, unmyelinated SP + sensory fibers are less abundant.^17^ In some cases, CGRP and SP are also co‐expressed. In the marrow space, large‐caliber vessels are frequently wrapped by sympathetic TH + fibers rich in varicosities, and like sensory terminals, sympathetic axons can dissociate from vasculature to terminate as free‐nerve endings in the bone marrow (Fig. 4 *A*, *D*).^10^, ^17^

Systematic analysis of fiber types innervating bone compartments has been estimated by the number or total length of fibers normalized to tissue area or volume, respectively, of periosteum, mineralized bone, and marrow space of the mouse femur.^10^, ^15^ By fiber number in the distal femur, sensory axons densely innervate the periosteum (177.4 CGRP + sensory fibers/mm^2^, 154.1 NF200 + myelinated sensory fibers/mm^2^) and are markedly less abundant in the mineralized bone (15.9 CGRP + fibers/mm^2^, 7.6 NF200 + fibers/mm^2^) and bone marrow (9.9 CGRP + fibers/mm^2^, 3.3 NF200 + fibers/mm^2^).^10^ Sympathetic fiber density is likewise greatest in the periosteum (128.4 TH‐positive fibers/mm^2^), though less abundant than sensory fibers. In contrast to sensory innervation of deeper bone compartments, sympathetic axons have a striking presence in mineralized bone (60.2 TH + fibers/mm^2^) and marrow (45.3 TH + fibers/mm^2^).^10^ These relative distributions are consistent with measurements of total fiber length for sensory and sympathetic fibers.^15^ Although these studies are powerful for comparing relative distribution of skeletal innervation, the high degree of fiber branching and winding through the tissue and the limited working depth in standard histological sections remain obstacles to quantifying the absolute number of neurons that innervate different bone compartments. Advances in tissue clearing techniques and whole‐mount preparations are beginning to be applied to bone that will improve quantitative mapping of skeletal innervation and secondary architecture such as glial cells.^54^, ^55^, ^56^

## Pathways to the Brain

There have only been a few studies that have mapped skeletal sensory input to and within the CNS. Retrograde labeled sensory neurons innervating rat tibial periosteum, medullary cavity, and trabecular bone have been observed in ipsilateral DRG spanning lumbar regions L_1_ to L_6_, but are concentrated in L_3_.^19^ Sensory neurons send their centrally projecting axons from the DRG to synapse with second order projection neurons or interneurons within distinct layers of the dorsal horn of the spinal cord. One study combined retrograde labeling from brain regions implicated in pain processing, with markers of neuronal activity in the spinal cord following noxious mechanical stimulation of bone, to further identify central pathways that may transmit information about bone pain toward the brain.^57^ They found that acute noxious mechanical stimulation of the rat tibial diaphysis activated neurons in the superficial dorsal horn (laminae I and II) that projected to the lateral parabrachial nucleus (LPB), but not the ventroposterolateral (VPL) or gracile nucleus. This suggests that painful stimuli in the bones of at least the lower limbs of rats are conveyed through the spinoparabrachial pathway (SPB), but not the spinothalamic tract (STT) or the postsynaptic dorsal column (PSDC), respectively. Interestingly, the spinoparabrachial pathway carries a broad range of nociceptive inputs from diverse tissues, and the lateral parabrachial nucleus further connects with brain regions implicated in emotional aspects of pain and homeostatic adaptation in response to pain.^58^ Only a single study has attempted to identify cortical somatosensory projections of sensory inputs derived from bone.^59^ It showed that electrical stimulation of nerves that enter the bone marrow of the cat humerus generates evoked potentials in topographically relevant areas of the primary and secondary somatosensory cortices.

Central sympathetic networks influencing bone were recognized when it was discovered hat leptin‐deficient mice have high vertebral trabecular bone mass.^60^ Follow‐up studies revealed that leptin acts as an anti‐osteogenic signal mediated centrally by glucose responsive neurons in the ventromedial hypothalamus (VMH) that is relayed peripherally through sympathetic pathways.^27^ A hierarchical circuit controlling sympathetic innervation of rat femoral epiphyseal bone marrow has also been inferred by multisynaptic tract‐tracing.^61^ Several autonomic projection pathways from the brainstem and the hypothalamus demonstrated connections to the femur via a sympathetic relay through preganglionic neurons concentrated in the intermediolateral cell column (IML) of the spinal cord (lower thoracic and upper lumbar segments T_4_ to L_1_) and postganglionic neurons in paravertebral chain ganglia (lumbar levels). Central networks labeled by this approach collectively illustrate that the bone does not appear to be innervated by unique cell groups, but rather is regulated by circuitry common to sympathetic control of other peripheral targets—likely in part due to shared mechanisms underlying autonomic mediation of vascular tone.

Together, these few functional and anatomical studies have only just begun to identify the central projection pathways that carry signals to and from the bone to brain and back again. Expanding the map of the brain regions involved in coding sensory signals as they pass toward the brain, and controlling efferent signals to bone, will be invaluable to understanding bone pain and neural regulation of bone metabolism.

## Neural Regulation of Skeletal Development, Homeostasis, and Repair

Neural involvement in endochondral ossification during skeletal development was inferred as early as 1925 by Fernando de Castro. At the site of endochondral ossification, de Castro detected nerve fibers terminating near osteoblasts that atrophied upon calcification, suggesting a precise spatial and temporal influence of nerves in skeletal development (referenced in Sherman^62^). In the mouse and rat, neurons appear first in the perichondrium of the diaphysis almost simultaneous with the first signs of mineralization.^29^, ^42^, ^50^, ^63^ Nerves accompanying blood vessels invade through the cartilage canals to reach the primary, and later the secondary, ossification centers, where they maintain a close relationship to the bone‐cartilage interface and the endosteum, sites of high osteogenic activity. At each site of bone formation, the appearance of sensory nerve fibers coincides with vascularization and ossification, and precedes the appearance of sympathetic nerve fibers,^50^, ^63^ hinting at a predominant role of the sensory nervous system in bone development. Ablation of sensory innervation by genetic or pharmacological approaches does not inhibit bone development, but consistently results in decreased bone mass in adult mice,^47^, ^64^, ^65^ suggesting that sensory nerves contribute to skeletal homeostasis and mineralization during development.

Bone readily adapts to functional demands in response to mechanical strain to maintain the structural integrity of the adult skeleton. It is clear that local skeletal cells sense and transduce mechanical stimulation to drive adaptive remodeling^66^; however, this does not fully account for bone formation observed at sites far from the region of maximal strain. For example, in the male rat, ulnar end‐loading induces bone formation in the loaded bone, as well as in the unloaded contralateral and adjacent axial bones.^67^ Brachial plexus anesthesia during loading, which temporarily blocks nerve activity in the sensory, sympathetic, and motor neurons that innervate the forelimb, significantly diminishes adaptive bone modeling in the loaded ulna and completely abolishes adaptive mineral deposition at distant sites.^67^ This may be due to interneuron connections within the spinal cord to sensory neurons targeting the contralateral limb and hind limbs^68^; sensory neuron interconnectivity between limbs is understudied, but provides an interesting avenue for future work. Preliminary evidence further suggests that mechanical loading increases synaptic connections between limbs^68^ and induces periosteal nerve sprouting.^55^ Thus, sensory neurons in bone are poised to detect, potentiate, and propagate adaptation to mechanical loads.

Nerves also dynamically rearrange to innervate bone tissue following fracture, in a sequence reminiscent of development. Following fracture, the appearance of sprouting fibers into areas of chondrogenesis precedes vascularization and ossification. New or regenerating GAP43 + axons robustly sprout into the fracture hematoma and the surrounding hypertrophic periosteum.^69^ Numerous CGRP + and SP + sensory fibers emerge from the deep layers of the periosteum to terminate as fine, varicose endings in the cartilage callus and penetrate newly formed woven bone around the fracture site.^70^, ^71^, ^72^ Neuropeptide Y–positive (NPY +) autonomic fibers are initially diminished at the fracture site, but exhibit sprouting during the inflammatory phase and the bone remodeling phase late in fracture healing.^73^ As healing progresses, many nerve fibers withdraw. In contrast, disordered and exuberant sprouting of sensory and sympathetic fibers into the marrow, mineralized bone, and periosteum remain in excess of 80 days in a non‐healed fracture, characterized by persistent fracture callus and nonunion, and aberrant mineralization in the marrow space adjacent to pathological nerve sprouting.^74^ Nerves are likely recruited to fracture sites to promote healing, both through release of vasoregulatory and osteogenic factors and through adaptive pain signals that prevent further injury.

## Molecular Signals From Neurons to Bone—Neurotransmitters

Nerves in bone integrate central relays controlling vascular tone and pain with peripheral regulation of skeletal cells such as osteoblasts and osteoclasts. These diverse functions utilize the same neurons and the same neurotransmitter pathways, facilitating simultaneous control of multiple aspects of skeletal homeostasis. True synapses have not been found within the bone.^75^ Instead, nonsynaptic vesicular fusion in axon varicosities releases signaling chemicals into the extracellular space that diffuse to target‐cell receptors by volume transmission. Ultrastructural analysis has provided evidence of free varicose nerve fibers in the vicinity of putative skeletal target cells: peptidergic sensory fibers running along the periosteal cellular layer and at the osteochondral junction at sites of mineralization, contacting osteoblasts and their precursors, osteoclasts, hematopoietic cells, and endothelial cells of intramedullary blood vessels^17^, ^75^, ^76^ and sympathetic nerve fibers contacting osteoblasts, osteoclasts, and bone marrow adipocytes.^27^, ^75^, ^77^ Several lines of evidence have teased out the influence of key neurotransmitters—the neuropeptides CGRP, SP, and NPY and the biogenic amine NE. Throughout this discussion of the effects of neurotransmitters on bone, it is important to consider that many of these can be expressed by other, non‐neural, cell types in bone. It is also worth cautioning against overinterpretation of in vitro effects on isolated bone cells, as receptors for these neurotransmitters are often expressed by a wide variety of bone cells as well as vascular and inflammatory cells, and thus this approach may not always translate in vivo.

### α‐CGRP

α‐CGRP, referred to from here simply as CGRP, is a sensory neuropeptide expressed throughout the central and peripheral nervous systems.^78^ CGRP is a potent vasodilator and is an important molecule in pain transmission and sensitization and inflammation.^79^, ^80^, ^81^ CGRP signals through a G‐protein coupled receptor (GPCR) complex of calcitonin receptor‐like receptor (CALCRL) and receptor activity‐modifying protein 1 (RAMP1).^82^ In vitro, CGRP is osteoanabolic with potential for direct and indirect inhibition of catabolism. CGRP increases proliferation and reduces apoptosis of osteoprogenitors^83^ and enhances osteogenic gene expression and osteoblast activity.^84^ Osteogenic consequences of CGRP are mediated by cAMP/PKA^85^, ^86^ through CREB1 activation of osterix^87^ and through activation of Wnt/β‐catenin.^83^, ^88^ CGRP may also promote osteoblast differentiation indirectly by inducing osteogenic signals from endothelial cells.^89^ High concentrations of CGRP suppress osteoclast maturation and activity in vitro,^90^, ^91^, ^92^ and CGRP regulates osteoblast production of RANKL and osteoprotegerin (OPG) to indirectly modulate osteoclastogenesis.^84^, ^85^, ^93^, ^94^

Of the sensory neuropeptides identified in bone, CGRP is the most well studied in vivo, with consistent osteoanabolic effects and some evidence for a neural origin. CGRP‐deficient mice exhibit reduced bone formation and decreased trabecular bone mass with no change in cortical bone or strength.^95^ The mice in this latter study had decreased CGRP reactivity in the DRG and loss of CGRP + nerve fibers in the bone, suggesting at least some of this effect might be of neural origin, but this remains to be confirmed. Conversely, mice overexpressing CGRP under the control of the osteocalcin promoter have increased trabecular bone formation and density^96^; of interest, calvarial cultures from osteocalcin‐CGRP mice produced CGRP, whereas cultures from nontransgenic mice did not, suggesting an alternative endogenous source for this neuropeptide. Activation of cells expressing TRPV1 suppresses alveolar bone resorption by a mechanism including osteoclast suppression by CGRP.^91^ CGRP promotes bone accrual during fracture repair.^87^ Magnesium rod implant into the medullary cavity of rat femur induces cortical bone formation and accelerates fracture healing.^87^ Mechanistically, magnesium induces CGRP release from local sensory fibers that in turn stimulates osteogenic differentiation of periosteum‐derived stem cells.

### SP

SP is a member of the tachykinin peptide hormone family encoded by the *tachykinin precursor 1* (*TAC1*) gene. SP is expressed in the brain and peripheral nerves and is often co‐released with CGRP; thus, it is involved in many overlapping processes including vasodilation, inflammation, and pain.^97^, ^98^ SP is also broadly expressed in non‐neural tissues including macrophages and neutrophils, epithelial cells, and endothelial cells,^99^, ^100^ and SP secretion from osteoblast‐ and osteoclast‐lineage cells has been detected in vitro.^101^, ^102^ SP binds and activates neurokinin‐1 receptor (NK‐1R), expressed in osteoblasts, osteocytes, and osteoclasts, as well as their precursors.^103^, ^104^, ^105^ In vitro, SP promotes viability and proliferation of osteoblast precursors and stimulates osteogenesis, in part through MAPK‐Erk and Wnt/β‐catenin signaling.^101^, ^105^, ^106^, ^107^ SP can drive osteoclastogenesis directly by inducing nuclear translocation of NF‐κB in osteoclast precursors, independent of RANKL, and indirectly by stimulating RANKL production in osteoblast lineage cells.^105^ In vivo studies of SP have shown differential effects on bone metabolism dependent on the bone under study and the physiological status of the bone.^98^, ^108^, ^109^ It is perhaps particularly difficult to isolate the effects of SP on bone cells because of its pervasive role in inflammation and its broad expression profile. Tissue‐specific genetic manipulation of SP and its receptor would be beneficial to resolve the roles of SP on bone metabolism and bone pain as detailed in the following section under Bone Pain.

### NE

NE is the canonical neurotransmitter of postganglionic noradrenergic sympathetic nerves. Adrenergic receptors (ARs) including β_1_, β_2_, α_1B_, α_2A_, α_2B_, and α_2C_, have been detected in osteoblast lineage cells,^27^, ^110^, ^111^, ^112^, ^113^, ^114^ and β_2_, α_2A_, α_2B_, and α_2C_ are expressed in osteoclast lineage cells.^111^ There is an abundance of pharmacological agents targeting ARs owing to their therapeutic value against life‐threatening conditions such as hypertension, asthma, and cardiac arrest.^115^ Thus, these drugs have been exploited to great lengths for mechanistic studies into noradrenergic influence on bone and the potential for their use to improve bone mass, and are thoroughly reviewed elsewhere.^116^ The effects of sympathetic signaling through NE have been extensively studied following the discovery of the central leptin‐sympathetic outflow‐bone axis (reviewed in Elefteriou and colleagues^117^). Briefly, bone mass is increased by genetically disrupting the noradrenergic signaling axis, either by knockout of the NE‐synthetic enzyme dopamine β‐hydroxylase or β_2_‐AR.^27^, ^118^ Mechanistically, sympathetic activity induces osteopenia as a result of activation of the β_2_‐AR on osteoblasts, which suppresses osteoblast proliferation and promotes release of RANKL to support osteoclastogenesis.^27^, ^113^, ^118^ Although chronic stimulation of sympathetic outflow may have detrimental effects on bone, β_2_‐AR signaling in osteoblasts and osteocytes also disrupts their capacity to maintain the endosteal hematopoietic stem/progenitor cell (HSPC) niche; and thus, upon physiological activation can positively regulate hematopoiesis.^110^, ^119^ In the context of cancer, both progression and metastasis are associated with chronic stress, a condition that stimulates sympathetic outflow.^120^, ^121^, ^122^ In bone metastasis, sympathetic signaling via activation of β_2_‐AR on osteoblasts leads directly to cancer cell recruitment and indirectly to vascular changes that promote colonization of metastatic cancer cells in the bone marrow.^121^, ^123^, ^124^ Last, NE uptake by bone and immune cells may also contribute to extraneuronal control of local NE homeostasis and actions on the skeleton.^56^, ^125^, ^126^

### NPY

NPY is the most abundant neuropeptide in the mammalian CNS and is also released from peripheral sympathetic nerves with NE. NPY contributes to neural control of a wide variety of physiological processes including stress and anxiety, appetite, circadian rhythm, cardiovascular activity, immune function, and pain.^127^ Peripherally, NPY is a potent vasoconstrictor. In addition to neural sources of NPY, it is also found in the adrenal medulla, and *Npy* mRNA has recently been detected in calvaria and femur.^128^ NPY and its related peptides, peptide YY (PYY) and pancreatic polypeptide (PP), can act on several common receptors, Y1, Y2, Y4, Y5, and Y6, which all couple to G_i_ proteins resulting in decreased cAMP production upon activation.^127^ NPY acts both centrally and peripherally to influence bone metabolism as deletion of NPY results in increased bone formation that is only partially rescued by re‐addition of NPY to the hypothalamus.^129^ Central control of bone metabolism mediated by NPY is through an inhibitory effect on Y2 expressing neurons; hypothalamic deletion of Y2 receptors stimulates osteoblast activity and increases cortical and cancellous bone formation.^130^ Knockout or pharmacological antagonism of Y1 increases bone formation resulting in high bone mass, though this is attributed in part to Y1 expression in osteoblasts providing evidence of local control of bone metabolism by NPY.^131^, ^132^, ^133^ In vitro, Y1 is expressed in induced bone marrow mesenchymal stromal cells (BMSCs) and gene expression increases with differentiation into osteoblasts; it is not detected in bone marrow macrophages (BMMs),^128^ suggesting a predominant local action of NPY on osteoblasts.

A key feature of all neurotransmitters identified to date in bone is their reliance on GPCR‐mediated signaling. Specifically, neurotransmitters bind their cognate GPCRs to stimulate or inhibit second messenger signaling cascades, as determined by coupling to cytoplasmic G‐proteins. This modulates a variety of downstream responses including protein secretion, ion channel opening, and gene transcription. In this way, volume transmission of neuropeptides and neurotransmitters from peripheral sensory and sympathetic fibers has the potential to produce summative, wide‐reaching, and potentially long‐lasting effects on bone including maintenance of skeletal homeostasis, vasoregulation, and peripheral sensitization. Though challenging, more studies are needed to investigate the duality and cumulative effects of neural signals on bone and their context and location‐specific functions.

## Molecular Signals From Bone to Neuron—Growth Factors, Guidance Molecules, and Inflammatory Mediators

Recent work has interrogated the relationships between local cell‐derived factors, nerve function, and bone health. Three key molecules, and their impact on skeletal homeostasis through neural and non‐neural mechanisms, are discussed in the following sections.

### NGF

During skeletal development, osteochondral lineage cells express NGF at the surfaces of newly forming bone, sites of incipient TrkA + nerve invasion.^134^ A functional NGF‐TrkA signaling axis is critical for proper neurovascular invasion and subsequent bone accrual.^134^ Similarly, following axial compression, periosteal and endosteal cells from the osteoprogenitor lineage acutely upregulate expression of NGF, which is followed by transient nerve sprouting at the periosteum.^55^ Disruption of NGF‐TrkA signaling abrogates load‐induced bone formation and downstream Wnt/β‐catenin signaling in osteocytes, whereas exogenous NGF enhances bone adaptation. Periosteal mesenchymal osteoprogenitor cells are the only NGF + skeletal cells in rib, but NGF is induced in a variety of skeletal cells in the fracture callus that resolves to mirror naive bone by 6 weeks postfracture.^135^ In vitro, NGF mRNA is expressed in BMSCs, calvarial osteoblasts, and osteoblastic cell lines and is upregulated during proliferative growth, upon loading, and with the addition of proinflammatory cytokines,^40^, ^55^, ^134^, ^136^ supporting a role for skeletal NGF signaling at spatially and temporally innervated sites coincident with high bone turnover. Aside from the classical role of NGF in nerve outgrowth and innervation of target tissues during development, precisely how NGF‐TrkA signaling contributes to bone development, anabolism, and repair is unclear. NGF has been shown to regulate nerve sprouting, activation, and neuropeptide expression and release.^137^ Additionally, NGF may modulate the vasculature by inducing neural expression of the pro‐angiogenic vascular endothelial growth factor (VEGF),^38^ directly activating TrkA receptors on vascular endothelial cells to promote angiogenesis,^39^ or potentially even acting directly on osteoblasts.^40^ Finally, there is evidence that the majority of bone nociceptors express receptors for NGF,^15^, ^37^, ^46^, ^138^, ^139^, ^140^, ^141^, ^142^, ^143^, ^144^, ^145^, ^146^ and blocking signaling through NGF and its receptors reduces pain in animal models of skeletal pathology and prevents inflammation‐induced changes in activity and sensitivity of bone nociceptors. This suggests NGF signaling may also be relevant to afferent functions of sensory neurons in bone, including pain as detailed in the following section under Bone Pain.

### Semaphorin 3a

Semaphorin 3a (Sema3a) is a vital cue for neural migration and axon guidance during development.^147^, ^148^ Loss of Sema3a leads to gross bone and cartilage formation abnormalities, such as fusion of vertebral bones, rib duplications, and poor alignment of the rib‐sternum junction.^147^ Conversely, Sema3a derived from synapsin‐expressing neurons enhances bone formation during development.^65^ Sema3a is also expressed by osteochondral lineage cells at future sites of ossification prior to or coincident with invasion of vessels and nerve fibers.^149^ Sema3a can inhibit osteoclastogenesis and promote osteoblast differentiation. Exogenous Sema3a decreases bone loss following ovariectomy and accelerates bone regeneration after cortical bone injury.^150^ In the mandible neurotrophism, sympathetic signals stimulate release of NGF and maintain Sema3a expression in bone cells and resident immune cells; these factors balance sympathetic and sensory neuron infiltration.^151^ Together, this illustrates an emerging signaling network that includes nerves, bone cells, and immune cells which converge to influence bone homeostasis through local secretion and maintenance of Sema3a.

### Prostaglandin E2

Prostaglandin E2 (PGE2) is a lipid metabolite and the most abundant prostaglandin in the body. PGE2 is a vasodilator, a potent inflammatory mediator, and a neuromodulator that can sensitize peripheral sensory neurons, contribute to inflammatory pain, and inhibit sympathetic neuronal release of NE.^152^, ^153^, ^154^ Administration of PGE2 has long been known to regulate both bone formation and resorption, typically with an imbalance in favor of formation.^155^ PGE2's anabolic potential is largely attributed to stimulation of osteogenesis through PGE receptor 4 (EP4) in osteoprogenitors.^156^, ^157^ Recent work has suggested that the osteoanabolic properties of endogenous PGE2 are also mediated by skeletal neurons. Specifically, PGE2 secreted from osteoblasts at active sites of remodeling signals through EP4 receptors in advillin‐expressing nerve fibers to promote bone formation.^64^ Loss of this signaling axis results in bone loss, whereas boosting PGE2 activation of advillin + nerves enhances bone regeneration following surgical ablation.^64^ Taken together, PGE2 signaling is suggestive of a novel converging mechanism for nerves to “sense” both bone pain and bone remodeling.

## Bone Pain

Pain is associated with most bony pathologies, and depending on the site of pathology, the quality of the resulting pain can be distinct. With fracture that involves the periosteum, pain responses are often described as splitting, excruciating, generally localized to the site of injury, and often radiating “up and down” throughout the body.^158^ By contrast, for pathology confined within the bone marrow, such as skeletal metastases, pain is often described as dull, diffuse, and difficult to localize. In some patients with osteoarthritis, resting bone pain has been found to be better correlated with intraosseous venous stasis than the presence of degenerative changes in the joint.^159^ Intraosseous varicose veins, or “bone perforators,” have also been reported to cause intense pain with qualities that combine both periosteal and marrow responses, likely due to their location, which spans the cortical bone.^160^ In addition to resting pain, patients with skeletal metastases or venous stasis often have bouts of intense “breakthrough” pain that are associated with movement or weight bearing.^161^, ^162^ Small‐diameter mechanosensitive sensory afferents covering the periosteal surface can be selectively activated by high‐threshold mechanical stimuli,^163^, ^164^ and Aδ nociceptors in bone marrow respond to high‐threshold intraosseous pressure,^22^ providing the physiological foundation for perceived pain in response to mechanical distortions. In addition, peripheral sensory endings in bone can be activated by growth factors NGF and the GDNF family of ligands, as well as mixture of inflammatory mediators including histamine, serotonin, bradykinin, and PGE2,^46^, ^164^, ^165^ and can directly contribute to pain associated with inflammation and bone metastases.

As noted above, both the periosteum and the bone marrow contain neurons that are capable of detecting and transmitting information about noxious stimuli. These can be enhanced during pathophysiologic states in multiple ways. First, local processes can induce sprouting of sensory neurons. From the perspective of bone healing, this may be adaptive as discussed for fracture where nerve recruitment may promote repair by augmenting protective pain detection as well as efferent anabolic cues. However, it can also contribute to increased pain. Metastatic bone tumors in particular are known to induce increases in the number of sensory fibers during tumor growth,^138^ and blockade of nerve fiber sprouting significantly decreases tumor‐associated pain in rodent.^139^ Beyond de novo sprouting, chronic, particularly inflammatory, processes can cause peripheral and/or central sensitization of skeletal nociceptors. Specifically, sensory nerves that innervate both periosteum and the bone marrow can be sensitized by a number of different inflammatory mediators, including NGF, artemin, neurturin, GDNF, histamine, serotonin, bradykinin, and PGE2.^46^, ^164^, ^165^, ^166^ Thus, even in the absence of a change in innervation, inflammation can elicit pain in response to normally innocuous stimuli (allodynia) or increase sensitivity to noxious stimuli (hyperalgesia). In many cases bone pain also presents as cutaneous sensitivity at sites surrounding or distant to the injury (referred pain).^167^, ^168^, ^169^, ^170^ This fact has been leveraged in preclinical studies for the relative ease of studying thermal and mechanical sensitivity of the skin near the affected bone as a surrogate for bone pain (reviewed in Nencini and Ivanusic^171^). However, preclinical studies that that use cutaneous sensitivity as a surrogate for bone pain may overestimate analgesic efficacy, impacting potential for clinical translational success. It is noteworthy that many preclinical studies are now incorporating alternative, more clinically relevant assays (eg, changes in weight distribution and activity levels) that better reflect the patient experience of bone pain and provide information about functional status.^46^, ^165^, ^166^, ^172^, ^173^, ^174^, ^175^, ^176^, ^177^

### Relationships between the nervous system, bone pain, and anabolism

It is interesting that the nerves that innervate skeletal tissues appear to function in both skeletal pain and anabolism, and influence a variety of different skeletal cell types. This implies that manipulating sensory or sympathetic neurons with drug therapies targeted to bone pain could have effects on anabolism and vice versa. A case to illustrate this is the emerging success story of the development of therapies directed against NGF signaling to treat bone pain. NGF‐TrkA signaling is an important mediator of both acute nociception and chronic pain.^178^ In rodents, inhibition of NGF‐TrkA signaling is effective in reducing pain associated with arthritis, fracture, and tumor growth.^37^ Tanezumab®, a humanized anti‐NGF antibody that blocks NGF‐TrkA and NGF‐p75 mediated signaling, is currently in phase III clinical trials for treatment of pain from osteoarthritis.^178^ During phase II trials in 2009, tanezumab was successful in treating pain, but patients within the high‐dose treatment groups developed rapidly progressive osteoarthritis,^179^, ^180^ a joint condition that resembles neurogenic arthropathy. Osteonecrosis and subchondral insufficiency fractures were also reported. This caused the drug to be placed on clinical hold. However, after modification of treatment regimens, including significant reductions in dose, tanezumab received fast‐track designation for treatment of chronic lower back pain and pain from osteoarthritis in 2017. In late 2018, Pfizer and Eli Lilly and Company reported the results from the first phase III study for patients with osteoarthritis treated for 16‐weeks.^181^ Pain management was significantly improved in the tanezumab groups and the incidence of rapidly progressing osteoarthritis was low (only 1.3%). Development of this therapeutic provides a modern‐day example of the relationship between neurotrophism and neurotrauma that harkens back to the days of Charcot and highlights the complex nature of interactions of the nervous system with skeletal tissues.

## Prospectus and Conclusions

The origins of the field of neuroskeletal biology were rooted in a great debate between the neurotrophic and neurotraumatic theorists. One hundred and fifty years later, the field has come together with the realization that nerves in bone can influence a number of important processes related to skeletal homeostasis, including anabolism and pain. Current research is evolving to consider both in order to define clinically relevant regulators of neuroskeletal health. The first success story is exemplified by the ongoing clinical trials of anti‐NGF therapeutics at doses that have been customized for both inhibition of pain with simultaneous maintenance of bone and joint health. However, more studies are needed to uncover the regulatory factors that mediate interactions between skeletal nerve fibers, bone cells, and other local cell populations, including vascular and inflammatory cells. It is important to recognize that many of the neural receptors and transmitters that might constitute druggable targets for a variety of disease processes are expressed by and signal in many of these different cell types in bone. Additional collaborations between neuroscientists and bone biologists will undoubtedly support identification of new interactions between bone and the nervous system, promoting the discovery and success of novel therapies.

## Disclosures

All authors state that they have no conflicts of interest.
